# Perceived emotional support mediates the association between childhood family adversity and adolescent mental health in the UK millennium cohort

**DOI:** 10.1038/s41598-025-26853-w

**Published:** 2025-11-28

**Authors:** Nicholas Kofi Adjei, Kenisha Russell Jonsson, Viviane S. Straatmann, Andrea Bellavia, Sanni Yaya, Ruth McGovern, Eileen Kaner, Ingrid Wolfe, David C. Taylor-Robinson

**Affiliations:** 1 Department of Public Health, Policy and Systems, University of Liverpool, Waterhouse Building 2nd Floor Block F, L69 3GL Liverpool, UK; 2 School of Public Health and Community Medicine, Institute of Medicine, Gothenburg University, Göteborg, Sweden; 3 Department of Public Health Sciences, Stockholm University, Stockholm, Sweden; 4 Department of Environmental Health, Harvard T.H. Chan School of Public Health, Boston, MA USA; 5 School of International Development and Global Studies, University of Ottawa, Ottawa, Canada; 6 Population Health Sciences Institute, Newcastle University, Newcastle, UK; 7 Department of Women and Children’s Health, King’s College London, London, UK

**Keywords:** Trajectories, Adversity, Poverty, Emotional support, Causal mediation analysis, Psychology, Risk factors

## Abstract

Childhood family adversity is associated with increased risk of developing mental health problems over the life course. We investigated how perceived emotional support in adolescence may mitigate the impact of childhood family adversity on adolescent mental health. We used longitudinal data from the UK Millennium Cohort Study on 9,269 children followed to age 17 years. Individuals were assigned to exposure trajectories characterised using group-based trajectory models that included measures of poverty and family dynamics up to age 14 years. Using counterfactual causal mediation analysis and four-way decomposition modelling approach, we evaluated how perceived emotional support at age 14 (measured using the three-item Short Social Provisions Scale) influences the association of childhood family adversity trajectories on mental health at ages 14 and 17, assessing the relative contributions of mediation and interaction simultaneously. Compared with children experiencing low family adversity and poverty, those exposed to childhood family adversity were almost three times more likely to experience poor mental health (RR 2·99, 95% CI 2 ·41 to 3·57) at age 14 and age 17 (RR 2·58, 95% CI 2·09 to 3·06). Perceived emotional support mitigates up to 18% (95% CI: 9% to 26%) of this effect at age 14, and 13% (95% CI: 5% to 22%) at age 17, mainly due to interactive mechanisms. Childhood family adversity has a strong effect on mental health, which is partially mitigated by emotional support in adolescence. Policies that support positive family functioning may be particularly beneficial for children who have experienced adversity.

**Supplementary Information:**

The online version contains supplementary material available at 10.1038/s41598-025-26853-w.

## Introduction

Adolescent mental health problems are becoming more common, affecting about 14% of young people globally^1^. In the UK, rising mental health problems amongst children and young people are of particular concern, with prevalence and inequalities rising dramatically in the wake of the COVID-19 pandemic^2^. Many studies suggest that poverty and social disadvantage are key determinants of adolescent mental health and well-being^3,4^. One pathway through which social disadvantage affects mental health is via exposure to childhood family adversity – harmful or stressful experiences occurring with the family environment during the early life. This include, but are not limited to, parental mental health, domestic violence, substance misuse and household poverty. In a recent study of a UK cohort, we showed that deprivation often co-occurs with and compounds other adversities, particularly parental mental illness, to amplify mental health problems in young people^5^.

In the absence of appropriate interventions or protective factors, evidence suggests that the co-occurrence or accumulation of risk from early childhood through adolescence can have lasting detrimental effects on both physical and mental health in later years^6^. While the relationship between childhood family adversity and poor adolescent mental health is well established^2,5^, we lack understanding of the complex interrelationships with protective factors at the macro and family level that can potentially mediate and/or moderate the impact of multiple adverse social circumstances.

Protective factors that build resilience to adverse socioeconomic conditions and family adversity may occur at different levels across the life course: the macro-level (e.g., environmental factors), community level (e.g., social cohesion and empowerment), family level (e.g., emotional and social support) and individual level (e.g., cognitive and personality development)^4^. The presence of these protective factors may improve outcomes for children growing up in disadvantaged circumstances. For instance, emotional support from family members and friends may improve not only social and cognitive skills but also resilience to cope with adverse conditions^7^. A number of studies have shown that psychosocial resources such as social and emotional support may have both direct and indirect protective effects on mental well-being in older adults^8^. However, the interplay of wider determinants and adversities, psychosocial resources and their impact on the mental health of young people have not been widely studied. Interventions to reduce the impact of social and family adversity are known to operate through various pathways^4^. Adolescence is a crucial period for individual development where adult patterns of mental illness may take root^3,4^, and thus family-level interventions during this time could serve as the main protective and enabling setting for children’s growth and mental wellbeing^4^. Yet, little is known about the pathways of family-related interventions that may offer the greatest opportunity to improve mental health outcomes for young people in the presence of social disadvantage and adversities.

Indeed, there is evidence that children growing up in disadvantaged socio-economic circumstances are differentially exposed to less family-level and emotional support^9^. Conversely, children may have differential susceptibility to adversity depending on the level of emotional support that they receive^10,11^. Understanding the contribution of differential exposure and susceptibility has potential implications for health inequalities policy to improve child health^11^. Furthermore, integrating both mechanisms in an analysis is crucial not only because it provides more insights into the underlying mechanisms, but also because a conventional mediation analysis may ignore exposure-mediation interactions, which can lead to potentially misleading conclusions^12^. In this current study, we build on our previous work on the clustering of family adversity and poverty^5^ to investigate how perceived emotional support in adolescence may mitigate the impact of childhood family adversity on adolescent mental health. To inform policy, we further assessed how differential exposure to, and differential susceptibility to perceived emotional support contribute to inequalities in young adolescents’ mental health. Identifying protective pathways are critical to understanding how the impact of risk factors can be modified or eliminated^11^. Thus, it is important to disentangle these pathways across different stages of the early life course in order to develop effective preventive mental health strategies and interventions.

## Methods

### Study setting and participants

Data are from the Millennium Cohort Study (MCS), a nationally representative cohort study of children in the UK born between September 2000 and January 2002, and followed up through seven survey waves. Parents were first interviewed when cohort members were approximately 9 months old, with subsequent interviews held at 3, 5, 7, 11, 14, and 17 years of age. Our sample includes data on singletons from wave 1 (9 months) to wave 7 (17 years). The respective numbers of responding families at each wave were 18,552, 15,590, 15,246, 13,857, 13,287, 11,726, and 10,625. At each wave, information was collected from the primary caregiver, usually the mother (about 99% at wave 1, 96% by wave 7). The MCS oversampled children from areas with relatively high proportions of ethnic minority populations in England and, in Wales, Scotland, and Northern Ireland, children from disadvantaged areas, by means of stratified clustering sampling design. Attrition in the MCS is socially patterned, with higher loss of follow-up among families in lower socioeconomic positions^13^. To reduce bias from selective non-response, we used wave-specific longitudinal non-response weights together with the stratified, clustered survey design. We applied these weights when constructing the exposure trajectories^5^ and in all regression models. Further details on weight derivation and sample design are provided in the MCS technical documentation^13^. The data collection of MCS is approved by the UK National Health Service Research Ethics Committee, and written consent was obtained from all participating parents at each survey; MCS1: South West MREC (MREC/01/6/19); MCS2 and MCS3: London MREC (MREC/03/2/022, 05/MRE02/46); MCS4: Yorkshire MREC (07/MRE03/32); MCS5: Yorkshire and The Humber-Leeds East (11/YH/0203); MCS6: London MREC (13/LO/1786). All methods were carried out in accordance with relevant guidelines and regulations. No additional ethical approval was needed for this secondary data analysis.

### Exposures

Six trajectory groups of poverty and family adversities (i.e., parental mental illness, domestic violence, and alcohol use) experienced by children aged 9 months to 14 years were previously established using group-based trajectory models^5^. These groupings were also used in the current study. The ‘low poverty and adversity’ group includes children with overall low exposure to childhood family adversities. The ‘persistent poverty’ group comprises children with a high likelihood of experiencing poverty throughout their childhood. The ‘persistent poor parental mental health’ group is mainly characterised by consistently high rates of poor parental mental health over time. The ‘persistent parental alcohol use’ and ‘persistent domestic violence’ groups consist of children exposed to parental alcohol use and domestic violence throughout their childhood, respectively. Finally, the ‘persistent poverty and poor parental mental health’ group includes children with high exposure to the co-occurrence of both persistent poverty and poor parental mental health throughout childhood (Figure S4). Description of measurements assessed for trajectory exposures can be found in Appendix S1.

### Mediator/effect modifier

Perceived emotional support was assessed at age 14 using the three-item Short Social Provisions Scale (SPS-3). The SPS-3 consists of three items assessing levels of emotional support: ‘*I have family and friends that help me feel safe*,* secure*,* and happy*’; ‘*There is someone I trust whom I would turn to for advice if I were having problems*’; ‘*There is no one I feel close to*’ (reversed ordered). Response categories ranged from 1 ‘*Very true*’, 2 ‘*Partly true*’, and 3 ‘*Not true at all*’. The individual items were summed to create a score that ranged from 3 to 9, with higher values indicating higher emotional support. The internal consistency of this measure was good in the study sample (Cronbach’s α = 0·69). The mediator was included as a binary variable, using a cut-off score of −1·25 below the normed mean score^14^ to define whether an adolescent had low perceived emotional support (coded as 0) or high emotional support (coded as 1) - set at the values (m = 7, 8 and 9), estimated as “optimum – higher” levels of perceived emotional support.

### Outcomes

Mental health was assessed using the Strengths and Difficulties Questionnaire (SDQ), which was completed by a parent or caregiver at ages 14 and 17 years. The SDQ is a validated psychometric tool that provides a dimensional measure of mental health in children and adolescents^15^. It consists of a 25-item questionnaire with five scales: hyperactivity, emotional symptoms, conduct disorders, peer problems, and prosocial behaviour. We used the total difficulties score (excluding prosocial behaviour items, with a score range of 0–40) to classify children into two groups and applied a validated cut-offs: ‘normal to borderline behaviour problems’ (0–16) and ‘socioemotional behavioural problems’ (17–40)^15^. The reliability and internal consistency of this measure were high at both age 14 (Cronbach’s α = 0·77) and 17 (Cronbach’s α = 0·83).

### Potential confounders

Confounders were selected based on their potential association with childhood adversity, emotional support and adolescent mental health^16^. These included child sex, lone parenthood, maternal education (degree plus, diploma, A-levels, GCSE A-C, GCSE D-G, none) and maternal ethnicity (white, mixed, Indian, Pakistani and Bangladeshi, black or Black British, or other ethnic groups) when the child was aged 9 months. The conceptual framework can be found in Figure S2 and Figure S3.

### Analysis

First, we described the sample characteristics by perceived emotional support, including the proportion of children exposed to childhood poverty and family adversity trajectories. We then estimated the prevalence of emotional support by exposure trajectories. Differences in prevalence were examined using Pearson’s Χ^2^ test.

**Fig. 1:**
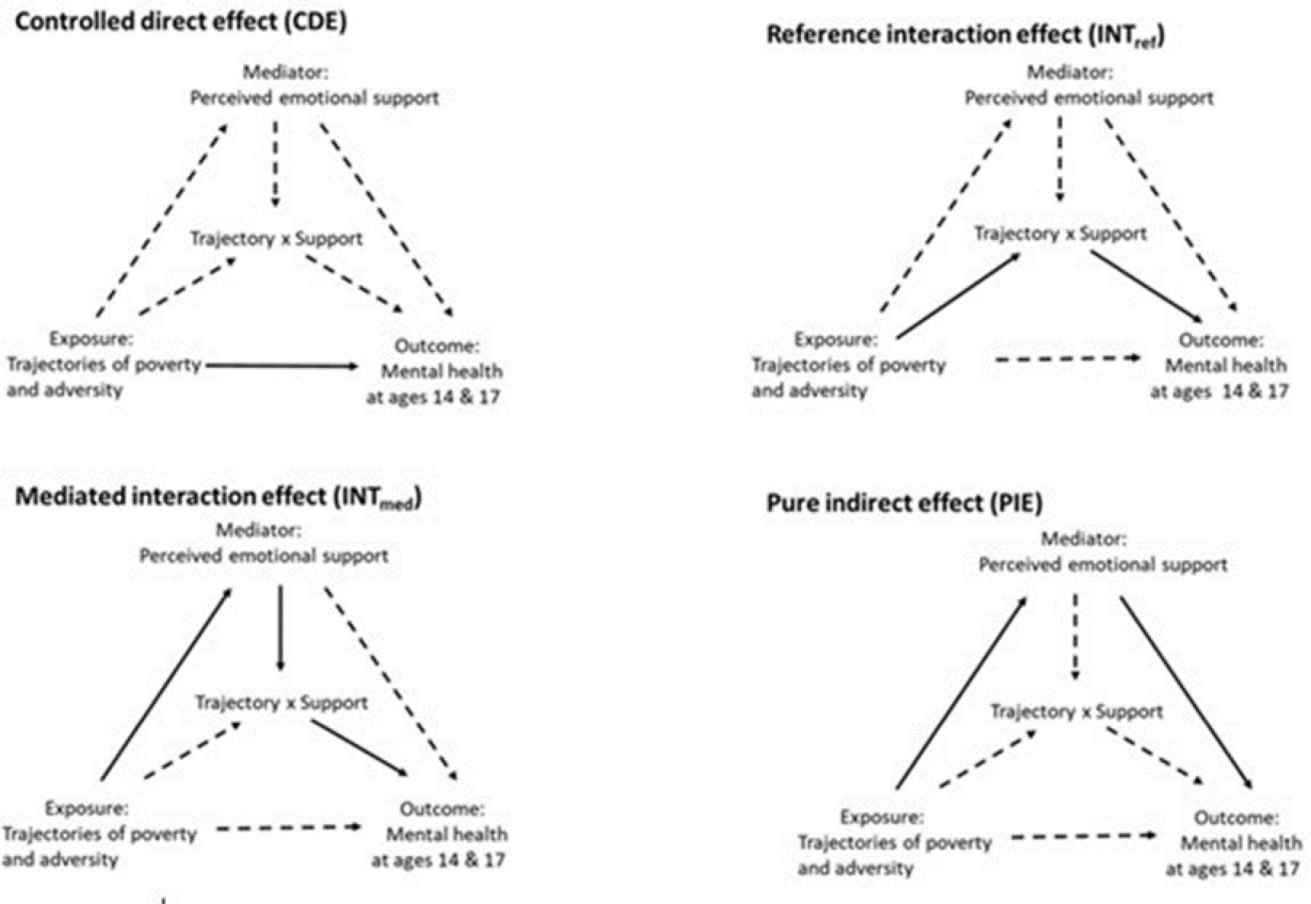
Causal diagram representing 4-way decomposition of the relationship between trajectories of poverty and family adversity, perceived emotional support and young people’s mental health.

Second, we used the counterfactual approach for causal mediation analysis to investigate the role of perceived emotional support at age 14 in the association between childhood family adversity and mental health at ages 14 and 17. The total effect of childhood family adversity on mental health was estimated in terms of Risk Ratio (RR), and we used the 4-way decomposition of the total effect to estimate mediating and interactive mechanisms^17^. Specifically, the total effect is divided into the following components: the controlled direct effect (CDE); reference interaction (INT_ref_); mediated interaction (INT_med_); and pure indirect effect (PIE) (see Table 1; Fig. 1 for more details).

**Table 1:** Formal definition and interpretation of the four-way decomposition of the total effect.

Effect	Counterfactual Definition	Interpretation
Controlled Direct Effect (CDE)	(*Y*_10_ − *Y*_00_)	The effect of poverty and family adversity on young people’s mental health when emotional support is set to be high (i.e. the effect due to neither to mediation nor interaction)
Reference Interaction (INT_ref_)	(*Y*_11_ − *Y*_10_ − *Y*_01_ + *Y*_00_)(*M*_0_)	The effect of poverty and family adversity on young people’s mental health due to the interaction between poverty and family adversity and emotional support that operates when emotional support would be high in the absence of poverty and adversity. (i.e. the effect only due to interaction)
Mediated Interaction (INT_med_)	*Y*_11_ − *Y*_10_ − *Y*_01_ + *Y*_00_)(*M*_1_ − *M*_0_)	The effect of poverty and family adversity on young people’s mental health due to the interaction between poverty and family adversity and emotional support and the fact that poverty and family adversity are causing low emotional support. (i.e. the effect due to both mediation and interaction)
Pure Indirect Effect (PIE)	(*Y*_01_ − *Y*_00_)(*M*_1_ − *M*_0_) = (*Y*_0*M*1_ − *Y*_0*M*0_)	The effect of poverty and family adversity on young people’s mental health through emotional support not accounting for the possible interaction between poverty and family adversity and emotional support. (i.e. the effect only due to mediation)

These components were then used to calculate the overall proportion eliminated (PE), which represents how much of the exposure’s effect can be mitigated by intervening on the mediator - from low to high emotional support. We also calculated differential exposure as the sum of the PIE and INT_med_, and differential susceptibility as the sum of INT_ref_ and INT_med_, as proposed by Diderichsen et al.^11^. Differential exposure indicates the extent to which inequalities in poverty and family adversity in young people’s mental health could be reduced by eliminating unequal exposure to perceived emotional support. Differential susceptibility indicates the proportion attributable to interaction. That is the extent to which inequalities in young people’s mental health could be reduced by eliminating the interaction between poverty, childhood family adversity and perceived emotional support.

We further repeated the analysis for the CDE of adversity on adolescent mental health when the mediator, emotional support, is set across the range of values observed in our sample (m = 3–9). To simplify interpretation, we also treated the exposure as a dichotomous variable, contrasting the low poverty and family adversity trajectory group (reference) vs. family adversity trajectory groups (i.e., poverty, parental mental illness, domestic violence and alcohol use). The output can be interpreted as the degree to which the excess risk in persistent poverty and family adversity, compared to low poverty and family adversity, would be eliminated or reduced following a hypothetical situation or scenario where children who received low perceived emotional support were provided with “optimum to high” levels of emotional support. We further estimated effects separately for the three main trajectory groups: poverty, parental mental health and exposure to parental mental health and poverty. The models were fitted with STATA 16.1 *med4way* package^18^·.

### Sensitivity analysis

Counterfactual causal mediation analyses rely on the assumption that: (i) there is no exposure-outcome confounding; (ii) there is no mediator-outcome confounding; (iii) there is no exposure-mediator confounding, and (iv) no mediator-outcome confounder is itself affected by the exposure^19^. Examples of potential unmeasured confounders may include maternal and paternal genetics/epigenetics. Hence, a sensitivity analysis is instrumental in testing whether unmeasured pre-exposure confounding could substantially change our conclusions. To assess the sensitivity of the initial findings to unmeasured confounding, a two-step process was followed^20^. First, we evaluated the change in each effect estimate resulting from the omission of each observed confounder, thereby providing a plausible range of bias. This is based on the assumption that the confounding impact of an unmeasured pre-exposure confounder is comparable to that of a measured confounder. Second, we corrected the bias from the original effect estimate and confidence interval (adjusted effect estimate = original estimate − bias). A change in the sign or precision of the effect after adjustment may lead to the conclusion that the initial results were sensitive to unmeasured confounding^20^. In addition, we assessed the robustness to unmeasured confounding using the E-values approach particularly for the CDE at age 14^21^ (Figure S6).

## Results

### Study population characteristics

Of the 10,625 participants at age 17 (wave 7), 9,269 were included in the final analytic sample (see Figure S1 for details). The main models were estimated using a complete-case analysis (i.e., participants with complete observations for the exposure trajectories, mediator, outcomes, and all covariates; *N* = 8,666). Table S1 shows a comparison of baseline sociodemographic and exposure variables between the MCS cohort at 9 months (*N* = 18,552) and the analytic sample. Distributions are broadly similar. Table 2 summarises the characteristics of the cohort members by perceived emotional support. Estimates derived using multiple imputation by chained equation (*n* = 25) are provided in Table S2. A higher proportion of adolescents who reported low perceived emotional support were in high adversity groups compared to those who reported high emotional support. We also observed differences in socioeconomic status and ethnicity. In Figure S5, we show the prevalence of low perceived emotional support by adversity trajectories, indicating that adolescents exposed to social adversity, either singly or in combination were more likely to report low perceived emotional support. For instance, the prevalence of low emotional support was 19.3% for adolescents in the persistent poverty and poor parental mental health trajectory group compared to 10.8% in the low adversity and poverty trajectory group (Figure S5).

**Table 2:** Baseline characteristics and trajectories by perceived emotional support, observed data, weighted sample.

Characteristics	Perceived emotional support	
	High (*n* = 8046)	Low (*n* = 1,223)
Trajectories of poverty and family adversity		
Low poverty and adversity	3749 (46.6%)	453 (37.0%)
Persistent alcohol use	680 (8.5%)	77 (6.3%)
Persistent domestic violence and abuse	269 (3.3%)	54 (4.4%)
Persistent poor mental health	954 (11.9%)	160 (13.1%)
Persistent poverty	1646 (20.5%)	300 (24.5%)
Persistent poverty and poor mental health	748 (9.3%)	179 (14.6%)
**Child’s sex**		
Boy	3819 (47.5%)	580 (47.4%)
Girl	3961(49.2%)	589 (48.2%)
Missing	266 (3.3%)	54 (4.4%)
**Maternal education**		
Degree plus	1717 (21.3%)	194 (15.9%)
Diploma	761 (9.5%)	94 (7.7%)
A-levels	804 (10.0%)	126 (10.3%)
GCSE A-C	2487 (30.9%)	386 (31.6%)
GCSE D-G	692 (8.6%)	119 (9.7%)
None	1304 (16.2%)	245 (20.0%)
Missing	281 (3.5%)	59 (4.8%)
**Maternal ethnicity**		
White	6494 (80.7%)	955 (78.1%)
Mixed	71 (0.9%)	13 (1.1%)
Indian	226 (2.8%)	31 (2.5%)
Pakistani and Bangladeshi	583 (7.3%)	107 (8.8%)
Black or Black British	244 (3.0%)	37 (3.0%)
Other ethnic groups	143 (1.8%)	25 (2.0%)
Missing	285 (3.5%)	55 (4.5%)

### Decomposition effects and comparing contributions

**Fig. 2:**
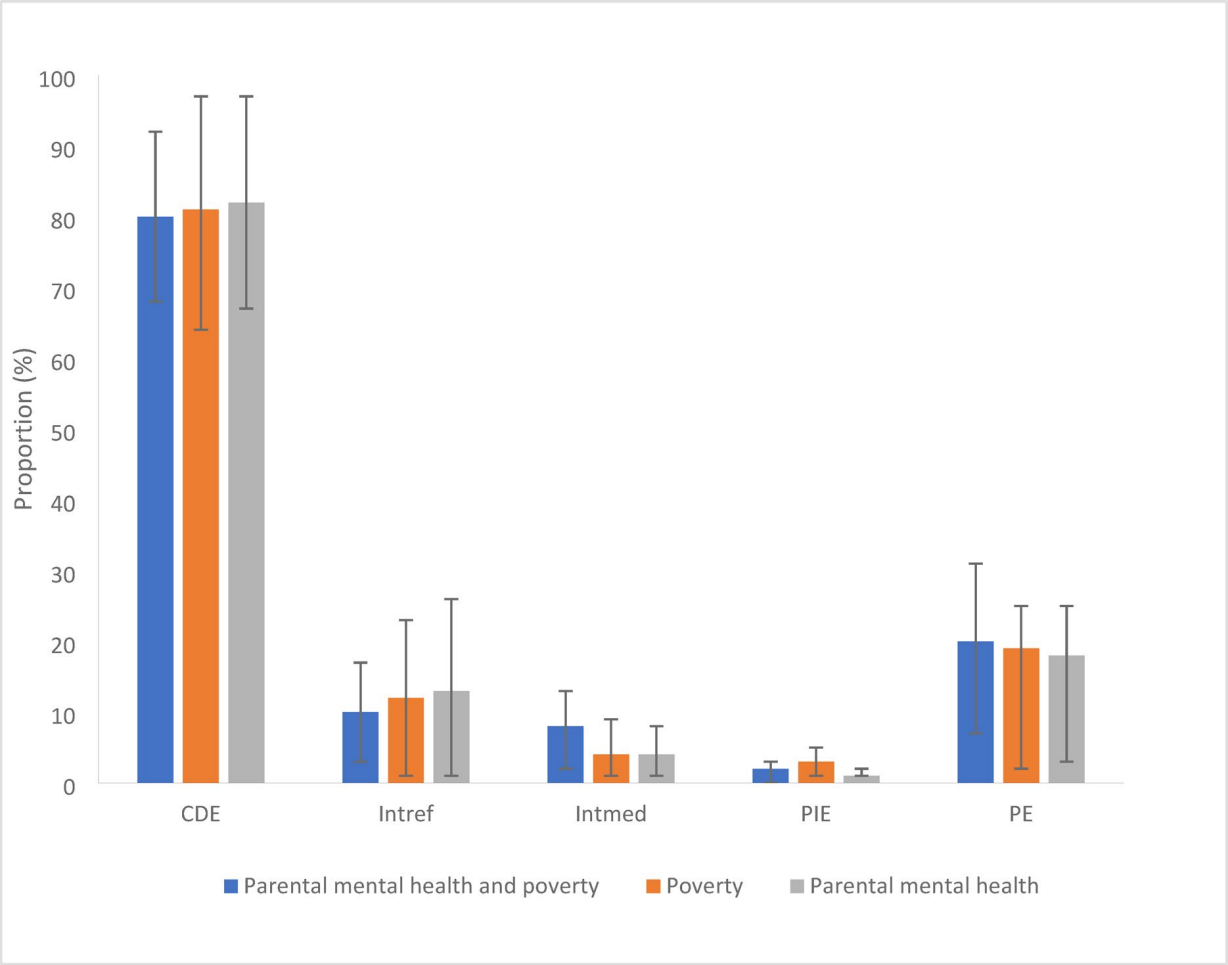
Proportion of the effect of poverty and parental mental health trajectories on young people’s mental health (age 14) due to mediation and interaction with perceived emotional support. Note: CDE = controlled direct effect (Due neither to mediation nor interaction); INTref = reference interaction (Due to interaction only); INTmed = mediated interaction (Due to mediation and interaction); PIE = pure indirect effect (Due to mediation only); PE = Proportional eliminated. Model adjusted for child sex, maternal education, ethnicity and lone parenthood.

Tables 3 and 4 shows the results from the effect decomposition. Compared with children who experienced low adversity and poverty, the adjusted relative risks of experiencing poor mental health among children and young people exposed to poverty and adversity were 2·99 (95% CI 2·41 to 3·57) at age 14 and 2·58 (95% CI 2.09 to 3·06) at age 17, respectively. At both ages, CDE (i.e., the proportion of total effect that would remain if all adolescents had higher levels of emotional support) accounted for 82% and 87% of the total effect estimates respectively, which was greater than the other three estimates for the decomposed effects. Nonetheless, there was a strong evidence for reference interaction (INT_ref_ = 13% at 14; INT_ref_ = 10% at 17). The combined PIE, INT_med_ and INT_ref_ (Proportion Eliminated) suggest that providing optimum emotional support would substantially reduce the risk of poor mental health; eliminating about 18% and 13% of the excess risk of mental health problems at age 14 and 17. The results were also comparable when assessing poverty and parental mental health trajectory groups either singly or in combination (Fig. 2). Differential susceptibility (sum of INT_ref_ and INT_med_) of perceived emotional support accounted for 17% and 13% of inequalities in young people’s mental health at age 14 and 17 years, respectively. We found little evidence of differential exposure (sum of PIE and INT_med_) (Tables 3 and 4).

**Table 3:** Proportion of the effect of trajectories of poverty and family on young people’s mental health (age 14) due to mediation and interaction with perceived emotional support, weighted sample.

Component	Risk ratio	(95% CI)
TE	2.99	(2.41 to 3.57)
Excess Relative risk (TE-1)	1.99	(1.41 to 2.57)

Four-way decomposition (excess relative risk)			Proportional Attributable	(95% CI)
CDE	1.63	(1.12 to 2.57)	82%	(73% to 90%)
INT_ref_	0.26	(0.10 to 0.41)	13%	(6% to 19%)
INT_med_	0.07	(0.02 to 0.14)	4%	(1% to 6%)
PIE	0.03	(0.01 to 0.05)	1%	(0% to 3%)
Total excess relative risk	1.99	(1.41 to 2.57)	100%	
**Proportion eliminated**, PE (INT_ref_ + INT_med_ + PIE)			18%	(9% to 26%)
**Differential exposure** (PIE + INT_med_)			5%	(1% to 7%)
**Differential susceptibility** (INT_ref_ + INT_med_)			17%	(8% to 27%)

Note: TE = total effect; Excess relative risk = proportion of the total effect (TE − 1) that can be attributed to each component in the four-way decomposition; CDE = controlled direct effect (Due neither to mediation nor interaction); INT_ref_ = reference interaction (Due to interaction only); INT_med_ = mediated interaction (Due to mediation and interaction); PIE = pure indirect effect (Due to mediation only); Mediator level in CDE has been set to the optimal value of emotional support (i.e., score ranged from 3 to 9); PE = Proportional eliminated. Model adjusted for child sex, maternal education, ethnicity and lone parenthood.

**Table 4:** Proportion of the effect of trajectories of poverty and family adversity on young people’s mental health (age 17) due to mediation and interaction with perceived emotional support, weighted sample.

Component	Risk ratio	(95% CI)
TE	2.58	(2.09 to 3.06)
Excess Relative risk (TE-1)	1.58	(1.09 to 2.07)

Four-way decomposition (excess relative risk)			Proportional Attributable	(95% CI)
CDE	1.37	(0.92 to 1.82)	87%	(78% to 95%)
INT_ref_	0.15	(0.03 to 0.27)	10%	(2% to 16%)
INT_med_	0.04	(0.01 to 0.08)	3%	(0% to 5%)
PIE	0.02	(−0.0 to 0.04)	0%	(−0% to 2%)
Total excess relative risk	1.58	(1.09 to 2.06)	100%	
Proportion eliminated, PE (INT_ref_ + INT_med_ + PIE)			13%	(5% to 22%)
Differential exposure (PIE + INT_med_)			3%	(1% to 6%)
Differential susceptibility (INT_ref_ + INT_med_)			13%	(5% to 23%)

Note: TE = total effect; Excess relative risk = proportion of the total effect (TE − 1) that can be attributed to each component in the four-way decomposition; CDE = controlled direct effect (Due neither to mediation nor interaction); INTref = reference interaction (Due to interaction only); INTmed = mediated interaction (Due to mediation and interaction); PIE = pure indirect effect (Due to mediation only); Mediator level in CDE has been set to the optimal value of emotional support (i.e., score ranged from 3 to 9); PE = Proportional eliminated. Model adjusted for child sex, maternal education, ethnicity and lone parenthood.

**Fig. 3:**
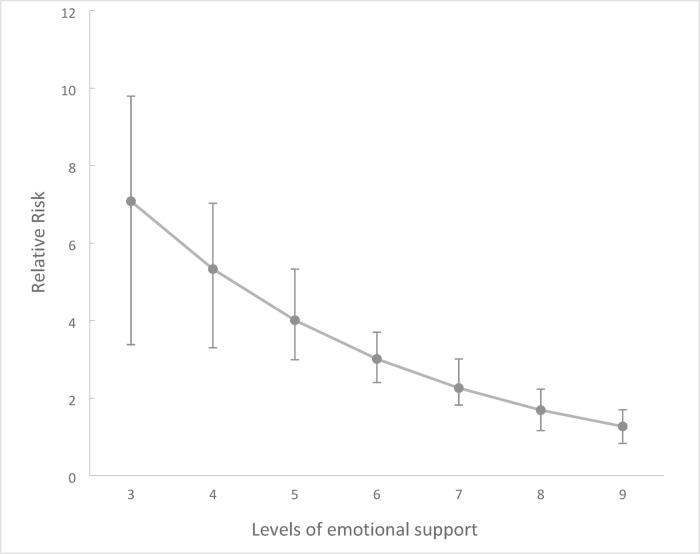
Controlled direct effect (CDE) of trajectories poverty and family adversity (exposure) on mental health at age 14 (outcome) when perceived emotional support (mediator) is fixed at values that range from the minimum to the maximum observed values.

In Fig. 3 we show the effect of poverty and adversity on adolescent mental health and how these changes depending on the level of perceived emotional support — from minimum to maximum (m = 3–9) as observed in the data. This illustrates the mitigating effect of emotional support, for instance, at age 14, if we had fixed the mediator (emotional support) at the highest level (m = 9), the negative impact of poverty and adversity on adolescent mental health would be reduced to RR 1.27 (95% CI 1·03 to 1·70). By contrast, for young people with the lowest level of perceived emotional support (m = 3), the risk ratio for the impact of adversity on mental health was 7·08 (95% CI 3·38 to 9·79) (Fig. 3).

## Discussion

In this study we assess the mitigating impact of perceived emotional support on the relationship between trajectories of poverty and childhood family adversity (i.e., parental mental illness, domestic violence, and alcohol use) and the mental health of young people. We used data from the UK Millennium Cohort Study and followed a large sample of children aged 9-months olds to 17 years. We show the harmful impact of family adversity across the childhood developmental stages on mental health in adolescence. We further demonstrate that perceived emotional support is an important mechanism through which cumulative poverty and childhood family adversities influence mental health among young people. We estimated how differential exposure to and differential susceptibility to perceived emotional support influence the impact of childhood family adversities on mental health. The results show that while children growing up in adverse circumstances experience less emotional support, the support they do receive is particularly beneficial for them. At age 14, approximately 18% of the total effect of childhood family adversity on adolescent mental health was mitigated by high emotional support. By age 17, the proportion eliminated was 13%. These findings suggest that emotional support may play a critical protective role, particularly in early adolescence, in buffering the mental health consequences of early-life family adversity.

To our knowledge, this study is the first to formally model and quantify the relationships between trajectories of family-related adversities during childhood, perceived emotional support, and mental health in early adulthood in a large population cohort with clear temporal ordering of the exposure, mediator and outcome. Indeed, there exist some studies that used social and emotional support as mediators to assess their protective effects against adverse health outcomes^22–24^, however, the longitudinal experience of children has yet to be captured over a longer period of time. Nonetheless, our findings align with previous cross-sectional studies, indicating that family and social support can mitigate the negative effects of social disadvantage on adolescent mental health^22,25^. Additionally, our results to some extent support previous findings that showed that emotional support is socially distributed^26^, where children and adolescents in socially disadvantaged circumstances are more likely to experience low perceived support^26^, which may negatively affect their mental health^8^.

The stark social inequalities in adolescent mental health illustrated in our study corroborate findings from other studies^27,28^. While the association between childhood family adversities and adolescent mental health is well understood^29,30^, we lack an understanding of the complex mechanisms or pathways connecting social conditions and experiences to mental health outcomes across the life course^28,31^. The potential pathways linking childhood family adversities and experiences to later mental health problems are still unclear but may involve a complex interplay between genetic, material, environmental, behavioural and social factors^31^. Since adolescence is the most vulnerable period for the development of mental health problems^28^, it is increasingly becoming clearer that other family-level pathways including psychological resilience factors may play a significant role in their development and well-being^28,32^. In this context, family stress or family process models are now gaining prominence in assessing family-based mechanisms through which social disadvantage influences children and adolescent outcomes, often focusing on parent-child relationships and parenting behaviours, including emotional support^32^. Our study is one of the first to decompose and compare the contribution of perceived emotional support to social inequalities in young people’s mental health using counterfactual mediation analysis; and we showed that larger proportions of social inequality in mental health outcomes may be mitigated or reduced by improving levels of emotional support.

Understanding the role of resilience factors in explaining social inequalities in adolescent mental health problems is critical to inform effective interventions^28^, and our study assessed the extent to which emotional support can mitigate the large social inequalities in adolescent mental health – whether this occurs via the process of differential exposure and/or differential susceptibility^11^. In other words, we consider differential exposure as the extent to which the total effect of family-related adversity on adolescent mental health is due to unequal exposure to emotional support and differential susceptibility the extent to which the total effect of family adversity on adolescent mental health is due to interaction between family adversity and perceived emotional support. Our results indicate that levels of emotional support are an important consideration in understanding social inequalities in adolescent mental health^23,25,28^, and this contribution is predominately due to the process of differential susceptibility or reference interaction. This suggests that lower levels of emotional support in adolescence exacerbate the effect of childhood family adversity on mental health problems in adulthood. On the other hand, we found minimal evidence for differential exposure or through mediation, indicating that analyses which only consider mediating pathways may underestimate the critical role of emotional support in the link between family-related adversity across childhood and mental health problems in adolescence. Our longitudinal evidence adds support to the notion that low family emotional support influence inequalities in adolescent mental health and well-being^33^, and explained in part why mental health disproportionately impacts children and adolescents who are socially disadvantaged^25^. Indeed, adolescents facing multiple childhood adversities often experience limited emotional support at home^16^. However, there is evidence that school-based socio-emotional learning programs and peer-support interventions can enhance resilience and psychological well-being in young people^34,35^. These supportive environments may help buffer the impact of adversity^35^, particularly when parents and caregivers are unable to provide consistent emotional care and support.

Before evaluating the implications of our findings, it is important to consider the limitations of the study. First, although we used modern methods for causal mediation analysis and adjusted for potential confounders based on previous research in this area^5,27^, we did not account for genetic and some environmental and interpersonal factors that could confound the complex relationships under investigation. While we interpret the findings cautiously, our sensitivity analysis - examining bias from unmeasured confounding - showed that the results are robust to omitted confounding (Table S3a to Table S4b). Second, although the trajectory groups – derived as latent classes from longitudinal models had high posterior probabilities, treating these latent constructs as observed variables may lead to underestimated standard errors and obscure uncertainty in group assignment. This limitation should be considered when interpreting the estimated effects. Third, even though we used validated measures and cut-offs for our variables of interest, they could be susceptible to reporting bias because they were based on self-reported information from parents and cohort members. Nevertheless, we believe that any potential bias may be low, as the scales showed good internal consistency. Fourth, issues related to missing data commonly arise in most longitudinal studies. This study however used attrition and longitudinal weights to handle non-random attrition and response bias. Despite these limitations, a key strength our study is that we utilised four different family-level adversities (i.e., poverty, parental mental illness, domestic violence, and alcohol use) that incorporate information on type and time of experienced adversity, using the most contemporary national representative UK birth cohort. The application of a counterfactual causal mediation analysis and the four-way decomposition modelling technique is also a strength of the study. This approach enabled us to estimate and assess the relative contributions of mediation and interaction simultaneously, and we found support for interactive effects. The method also offered a unique opportunity to assess the underlying mechanisms via the processes of differential exposure to and the differential effect of perceived emotional support, which may have important implication for policy^11^.

Our findings support other studies that advocate for strengthening a range of integrated and synergistic interventions to support families in order to address inequalities in child and adolescent health^28^. One important conclusion when differential susceptibility exists, as in our study, is that effective interventions to strengthen families’ capabilities to provide emotional support for children are likely to have a stronger mental health effect among the more susceptible^11^. That is to say that improving emotional and family support is a potentially levelling-up intervention. For example, parenting interventions which focus upon enhancing the parent-child relationship have been found to reduce aversive parenting practices, increase reflexive parental functioning and improve child and adolescent mental health outcomes^36,37^. Families who experience socioeconomic disadvantage have been found to benefit just as much as families without disadvantage in the short-term^38^. However, there is also evidence that stressful financial situations and family adversity may result in contexts wherein high levels of engagement may be difficult to achieve^39^. As such, family-level interventions which aim to enhance emotional support may form an important public health strategy to reduce health inequalities, when accompanied by ‘upstream’, structural and policy-level interventions to reduce child poverty and exposure to adversity^40^.

## Supplementary Information

Below is the link to the electronic supplementary material.

Supplementary Material 1

## Data Availability

The data that support the findings of this study are available from the UK Data Service by application, under license. For further information on how to obtain the dataset, visit the UK Data Service website (https://ukdataservice.ac.uk/).

## Abbreviations

CDE: controlled direct effect
INTmed: mediated interaction
INTref: reference interaction
MCS: Millennium Cohort Study
PE: proportional eliminated
PIE: pure indirect effect
RR: Risk Ratio
SDQ: Strengths and Difficulties Questionnaire
SPS: Social Provisions Scale
