# Supplementary material for: Perceived emotional support mediates the association between childhood family adversity and adolescent mental health in the UK millennium cohort
Source: Sci Rep. 2025 Nov 28;15:42730. doi: 10.1038/s41598-025-26853-w (PMC12663155; doi:10.1038/s41598-025-26853-w)
Supplement: Supplementary file 1 — Supplementary Material 1 [file 41598_2025_26853_MOESM1_ESM.docx]

**
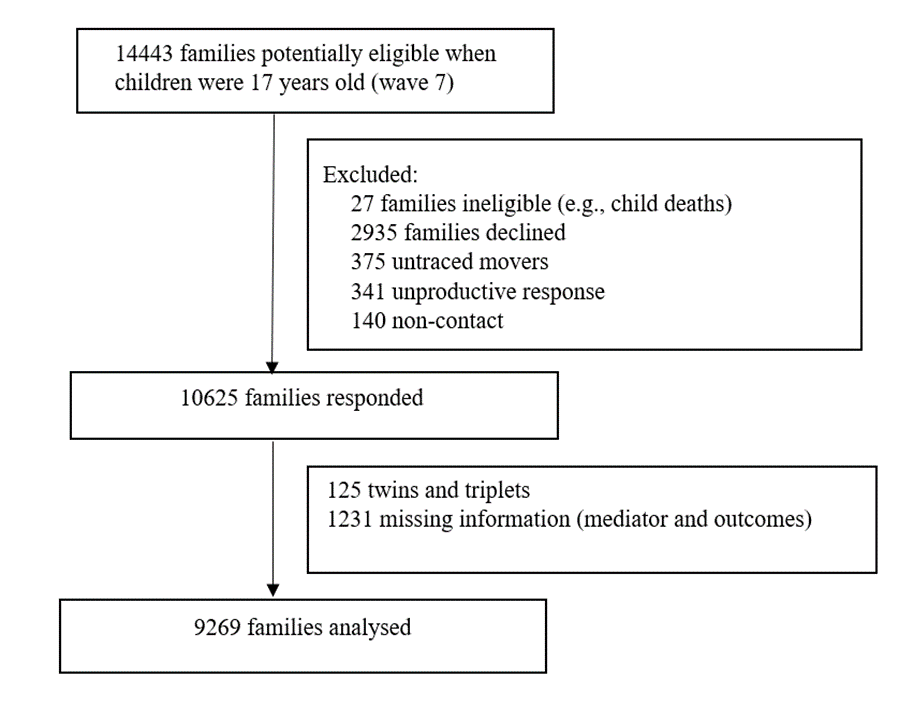
**

**Figure S1:** Study flow diagram showing inclusion and exclusion of cohort participant


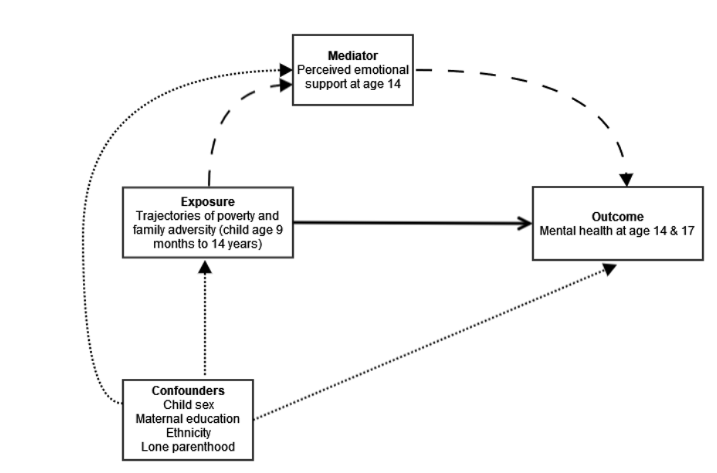


**Figure S2:** Logic model of the pathways from poverty and family adversity trajectory (child age 9 months to 14 years) to young people’s mental health (14 and 17 years), with direct pathway shown in bold, indirect pathways via the mediator in dashed lines and baseline confounding pathways in dotted lines


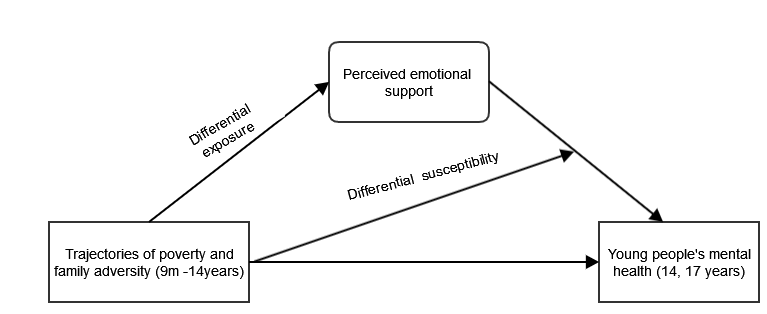


**Figure S3:** Conceptual model of the differential exposure and differential susceptibility to perceived emotional support, leading to inequalities in young people’s mental health by trajectories of poverty and family adversity. Confounders are included in the conceptual model.

**Appendix S1.** Description of measurements assessed for trajectory exposures

- **Parental mental ill health (Child aged 9 months)** – Rutter Malaise Inventory (RMI)^1^ scale was used to assess parental mental ill health· A shortened 9-item self-completed version of the RMI measuring depression, anxiety and psychosomatic illness was used· The 9-item short form included items ‘feel tired most of the time’, ‘feel miserable or depressed’, ‘worried about things’, ‘often get into violent rage’ ‘suddenly become scared for no good reason’, ‘easily upset or irritated’, ‘constantly keyed up or jittery’, ‘every little thing gets on nerves and wears you out’, and ‘heart race like mad’· Scores from these items were summed, and we used a validated cut off for mental ill health [‘yes (scores >=4)/no’]·
- **Parental mental ill health (Child aged 3 to 14 years) –** Kessler 6 (K6)^3^ scale was used to assess parental mental ill health in the last 30 days asking the responders how often they felt depressed, hopeless, restless or fidgety, worthless, or that everything was an effort· Respondents answered on a five-point scale from 1(all the time) to 5 (none of the time)· We reversed and rescaled all items from 0 to 4 for analysis purposes, so that high scores indicate high levels of psychological distress· We used a validated cutoff widely used in previous studies [‘yes (scores >=6)/no’]
- **Frequent parental alcohol use (Child aged 9 months to 7 years) –** the main responder answered a question about their usual frequency of alcohol consumption (‘*Every day, 5-6 times per week, 3-4 times per week, 1-2 per week, 1-2 per month, less than once a month or never’*)·

Dichotomised: [every day and 5-6 times per week (Yes) vs· 3-4 per week/1-2 per week/ 1-2 per month/never (No)]

- **Frequent parental alcohol use (Child aged 11 to 14 years) –** the main responder answered a question about the usual frequency of alcohol consumption *(‘>=4 times per week, 2-3 times per week, 2-4 times per month, monthly or less, or never’)·*

Dichotomised: [4 or more times a week (Yes) vs· 2-3 per week/2-4 per month/ monthly or less/never (No)]

- **Domestic violence and abuse (Child aged 9 months to 14 years) –** the main responder was asked about the use of physical force by the partner in relationship (‘*Yes, No’*)
- **Poverty (Child aged 9 months to 14 years)** – relative income poverty^4^, defined as household equivalised income of less than 60% of national median household income equivalised according to the Organisation for Economic Co-operation and Development (OECD) household equivalence scale.


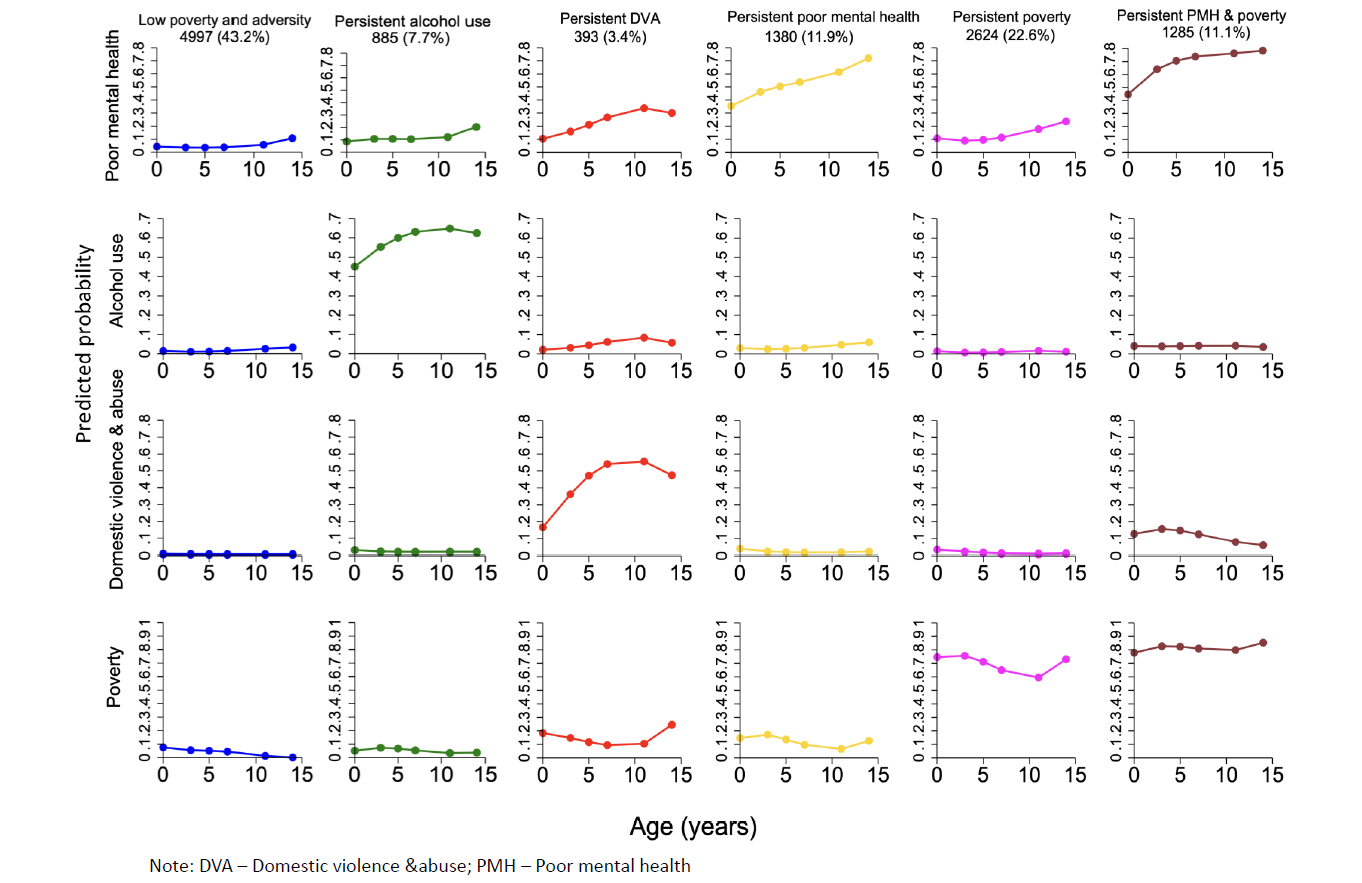


**Figure S4.** Estimated trajectory groups of family adversity and poverty in the UK Millennium Cohort Study. Excerpted from Adjei et al. 2022. *The Lancet Regional Health-Europe.^2^*

**Table S1.** Comparison of Millennium Cohort Study (MCS) baseline sample and the analytic sample, weighted

|  |  | |
| --- | --- | --- |
| **Characteristics** | **MCS sample at baseline (N=18,552)** | **Analytic sample (N=9,269)*** |
| **Exposures** |  |  |
| **Poverty** | 30.3% | 34.6% |
| Missing | 0.5% | 3.7% |
| **Poor parental mental illness** | 13.6% | 32.1% |
| Missing | 5.3% | 7.2% |
| **Domestic violence and abuse** | 3.6% | 3.2% |
| Missing | 26.3% | 29.5% |
| **Parental alcohol use** | 5.5% | 7.7% |
| Missing | 0.1% | 6.7% |
| **Child's sex** |  |  |
| Boy | 49.6% | 47.5% |
| Girl | 46.8% | 49.1% |
| Missing | 3.6% | 3.4% |
| **Maternal education** |  |  |
| Degree plus | 15.6% | 20.6% |
| Diploma | 8.3% | 9.2% |
| A-levels | 9.3% | 10.0% |
| GCSE A-C | 33.3% | 31.0% |
| GCSE D-G | 10.7% | 8.6% |
| None | 20.5% | 16.7% |
| Missing | 2.3% | 3.7% |
| **Maternal ethnicity** |  |  |
| White | 80.7% | 80.4% |
| Mixed | 0.9% | 0.9% |
| Indian | 2.5% | 2.8% |
| Pakistani and Bangladeshi | 6.6% | 7.4% |
| Black or Black British | 3.5% | 3.0% |
| Other ethnic groups | 1.8% | 1.8% |
| Missing | 4.0% | 3.7% |

Note: weighing variables: pttype2 (stratum variable), sptn00 (clustering at ward level), nh2 (finite population correction factor), survey weight ((aovwt2 (age 9 months, baseline), (fovwt2 (age 14)).

***** Exposures measured at age 14 (N=11,564). Further details on earlier waves are provided in Adjei et al. 2022. *The Lancet Regional Health-Europe*.^2^ Sociodemographic variables measured at age 17 (N=9,269).

**Table S2.** Baseline characteristics and trajectories by perceived emotional support, observed data, imputed data

|  |  |  |
| --- | --- | --- |
|  | **Perceived emotional support** | |
| **Characteristics** | **High (n= 8,046)** | **Low (n=1,223)** |
| **Child's sex** |  |  |
| Boy | 49.08% | 49.80% |
| Girl | 50.92% | 50.20% |
| **Maternal education** |  |  |
| Degree plus | 21.99% | 16.68% |
| Diploma | 9.74% | 8.01% |
| A-levels | 10.37% | 10.47% |
| GCSE A-C | 32.20% | 33.44% |
| GCSE D-G | 8.91% | 10.06% |
| None | 16.79% | 21.34% |
| **Maternal ethnicity** |  |  |
| White | 83.63% | 81.68% |
| Mixed | 0.91% | 1.14% |
| Indian | 2.93% | 2.70% |
| Pakistani and Bangladeshi | 7.47% | 9.08% |
| Black or Black British | 3.18% | 3.27% |
| Other ethnic groups | 1.88% | 2.13% |

Note: _Only percentages are presented for multiple imputed data as the_ *_ns_* _vary across the 25 imputed datasets. Imputation included all exposure, mediator, outcome and covariate variables._

**Figure S5.** Prevalence of low perceived emotional by poverty and family adversity trajectory

**Table S3a.** Sensitivity analysis: bias in each effect estimate due to removal of each observed pre-exposure covariate in the original model (14 years)

|  | Original | Bias Due to Removal of | | | |
| --- | --- | --- | --- | --- | --- |
|  |  | Maternal education | Ethnicity | Child's sex | Lone parenthood |
| Total effect | 2.99 | -0.7637 | 0.1333 | 0.0209 | 0.1573 |
|  | (2.41, 3.57) |  |  |  |  |
| Controlled direct effect | 1.63 | -0.5943 | 0.1163 | 0.0225 | 0.1547 |
|  | (1.12, 2.57) |  |  |  |  |
| Reference interaction | 0.26 | -0.0985 | 0.0131 | -0.0016 | 0.0023 |
|  | (0.10, 0.41) |  |  |  |  |
| Mediated interaction | 0.07 | -0.0629 | 0.0737 | -0.0003 | -0.0043 |
|  | (0.02, 0.14) |  |  |  |  |
| Pure indirect effect | 0.03 | -0.0084 | 0.0001 | 0.0003 | 0.0046 |
|  | (0.01, 0.05) |  |  |  |  |

**Table S3b.** Sensitivity analysis: adjusted point estimate and confidence interval with adjustment for an unmeasured pre-exposure covariate whose confounding role is comparable to each observed pre-exposure covariate (14 years)

|  | Original | Results after the removal of a bias associated with an unmeasured pre-exposure covariate whose confounding role is comparable to | | | |
| --- | --- | --- | --- | --- | --- |
|  |  | Maternal education | Ethnicity | Child's sex | Lone parenthood |
| Total effect | 2.99 | 3.75 | 2.86 | 2.97 | 2.84 |
|  | (2.41, 3.57) | (3.05, 4.45) | (2.31, 3.41) | (2.39, 3.54) | (2.27, 3.39) |
| Controlled direct effect | 1.63 | 2.23 | 1.51 | 1.61 | 1.48 |
|  | (1.12, 2.57) | (1.63, 2.83) | (1.03, 1.99) | (1.11, 2.11) | (1.02, 1.96) |
| Reference interaction | 0.26 | 0.35 | 0.24 | 0.26 | 0.26 |
|  | (0.10, 0.41) | (0.17, 0.53) | (0.09, 0.39) | (0.10, 0.41) | (0.10, 0.40) |
| Mediated interaction | 0.07 | 0. 13 | 0.07 | 0.08 | 0.08 |
|  | (0.02, 0.14) | (0.05, 0.22) | (0.02, 0.13) | (0.02, 0.14) | (0.02, 0.14) |
| Pure indirect effect | 0.03 | 0.04 | 0.02 | 0.03 | 0.02 |
|  | (0.01, 0.05) | (0.01, 0.07) | (0.01, 0.05) | (0.01, 0.05) | (0.01, 0.05) |

*Adjusted effect estimate = original estimate – bias

**Table S4a.** Sensitivity analysis: bias in each effect estimate due to removal of each observed pre-exposure covariate in the original model (17 years)

|  | Original | Bias Due to Removal of | | | |
| --- | --- | --- | --- | --- | --- |
|  |  | Maternal education | Ethnicity | Child's sex | Lone parenthood |
| Total effect | 2.58 | -0.6479 | 0.0298 | 0.0007 | -0.0174 |
|  | (2.09, 3.06) |  |  |  |  |
| Controlled direct effect | 1.37 | -0.5523 | 0.0259 | 0.0010 | -0.0094 |
|  | (1.02, 1.82) |  |  |  |  |
| Reference interaction | 0.15 | -0.0581 | 0.0030 | -0.0003 | -0.0061 |
|  | (0.03, 0.27) |  |  |  |  |
| Mediated interaction | 0.04 | -0.0330 | 0.0009 | 0.0001 | -0.0049 |
|  | (0.01, 0.09) |  |  |  |  |
| Pure indirect effect | 0.02 | -0.0045 | 0.0001 | 0.0001 | 0.0028 |
|  | (0.01, 0.04) |  |  |  |  |

**Table S4b.** Sensitivity analysis: adjusted point estimate and confidence interval with adjustment for an unmeasured pre-exposure covariate whose confounding role is comparable to each observed pre-exposure covariate (17 years)

|  | Original | Results after the removal of a bias associated with an unmeasured pre-exposure covariate whose confounding role is comparable to | | | |
| --- | --- | --- | --- | --- | --- |
|  |  | Maternal education | Ethnicity | Child's sex | Lone parenthood |
| Total effect | 2.58 | 3.23 | 2.55 | 2.58 | 2.59 |
|  | (2.09, 3.06) | (2.64, 3.81) | (2.07, 3.02) | (2.09, 3.07) | (2.09, 3.10) |
| Controlled direct effect | 1.37 | 1.92 | 1.55 | 1.37 | 1.38 |
|  | (1.02, 1.82) | (1.38, 2.46) | (1.07, 2.02) | (1.02, 1.82) | (1.02, 1.85) |
| Reference interaction | 0.15 | 0.20 | 0.15 | 0.15 | 0.16 |
|  | (0.03, 0.27) | (0.07, 0.35) | (0.03, 0.27) | (0.03, 0.27) | (0.03, 0.28) |
| Mediated interaction | 0.04 | 0.08 | 0.04 | 0.04 | 0.01 |
|  | (0.01, 0.09) | (0.02, 0.13) | (0.01, 0.08) | (0.01, 0.09) | (0.01, 0.09) |
| Pure indirect effect | 0.02 | 0.02 | 0.02 | 0.02 | 0.01 |
|  | (0.01, 0.04) | (0.01, 0.04) | (0.01, 0.04) | (0.01, 0.04) | (-0.01, 0.03) |

*Adjusted effect estimate = original estimate – bias

**Figure S6.** Sensitivity analysis for unmeasured confounding for CDE using E-values (14 years)

The minimum association to explain the RR of the CDE is 2.64 with a lower confidence limit (LCL) of 1.49. This means the unmeasured confounder would have to be associated with both adversity and mental health at age 14 by a RR of 2.6 times each. To move the LCL to include no effect, association of an unmeasured confounder would need to be 1.5 or above.

**References**

1. Connelly R, Platt L. Cohort profile: UK millennium cohort study (MCS). *International journal of epidemiology* 2014; **43**(6): 1719-25.

2. Nicholas Kofi Adjei DKS, Viviane S. Straatmann, Gabriella Melis, Kate M. Fleming, Ruth McGovern, Louise M. Howard, Eileen Kaner, Ingrid Wolfe, David C. Taylor-Robinson. Impact of poverty and family adversity on adolescent health: a multi-trajectory analysis using the UK Millennium Cohort Study. *The Lancet Regional Health-Europe* 2022; **13**: 100279.

3. Whitmore K, Tizard J, Rutter M. Education, Health, and Behaviour: Psychological and Medical Study of Childhood Development: Wiley; 1970.

4. Wickham S, Whitehead M, Taylor-Robinson D, Barr B. The effect of a transition into poverty on child and maternal mental health: a longitudinal analysis of the UK Millennium Cohort Study. *The Lancet Public Health* 2017; **2**(3): e141-e8.
